# 3D Texture Analysis in Renal Cell Carcinoma Tissue Image Grading

**DOI:** 10.1155/2014/536217

**Published:** 2014-10-09

**Authors:** Tae-Yun Kim, Nam-Hoon Cho, Goo-Bo Jeong, Ewert Bengtsson, Heung-Kook Choi

**Affiliations:** ^1^Department of Computer Engineering, Inje University, Injero 197, UHRC, Gimhae, Gyeongnam 621-749, Republic of Korea; ^2^Department of Pathology, Yonsei University, Seoul 120-749, Republic of Korea; ^3^Department of Anatomy, Gachon University, Incheon 406-799, Republic of Korea; ^4^Centre for Image Analysis, Uppsala University, 75105 Uppsala, Sweden

## Abstract

One of the most significant processes in cancer cell and tissue image analysis is the efficient extraction of features for grading purposes. This research applied two types of three-dimensional texture analysis methods to the extraction of feature values from renal cell carcinoma tissue images, and then evaluated the validity of the methods statistically through grade classification. First, we used a confocal laser scanning microscope to obtain image slices of four grades of renal cell carcinoma, which were then reconstructed into 3D volumes. Next, we extracted quantitative values using a 3D gray level cooccurrence matrix (GLCM) and a 3D wavelet based on two types of basis functions. To evaluate their validity, we predefined 6 different statistical classifiers and applied these to the extracted feature sets. In the grade classification results, 3D Haar wavelet texture features combined with principal component analysis showed the best discrimination results. Classification using 3D wavelet texture features was significantly better than 3D GLCM, suggesting that the former has potential for use in a computer-based grading system.

## 1. Introduction

Renal cell carcinoma (RCC) is the most common malignancy that arises in the adult kidney, representing 2% of all malignancies and 2% of cancer-related deaths. It is a histopathologically heterogeneous disease, subdivided into clear, papillary, granular, spindle, and mixed cell variants based on cytoplasmic features. The prognosis for RCC is based on tumor staging and histological grading [1]. Our four-stage grading system has been based on the papillary tumor grading and TNM staging system [2, 3].

Grading is a classification system for the progress of the cancer based on the degree of abnormality of the cancer cells. It plays an important role in clinical therapy decisions because it indicates a probable growth rate, the metastasis trends of the cancer, and other important information. Various grading systems have been proposed for RCCs, using nuclear, cytoplasmic, and architectural features. The available evidence suggests that nuclear grading is a better prognostic indicator than the other types of grading scheme. Skinner et al. were the first to propose a grading system based on nuclear morphology [4]. In 1982, Fuhrman et al. simplified Skinner et al.'s grading system, and many researchers have since then used this new classification system. Fuhrman et al.'s system is also a four-grade system, based on the size, shape, and contents of the tumor cell nuclei [5, 6].

Conventional grading, using visual observation, is prone to a degree of observer bias. Various grading systems have been proposed for RCCs, using nuclear, cytoplasmic, and architectural features. The available evidence suggests that nuclear grading is a better prognostic indicator than the other types of grading scheme. Even when the same grading system is used, different analysts may have different opinions, resulting in a possible interobserver problem or intraobserver problem. The interobserver problem refers to systematic differences among the observers' opinions. The intraobserver problem refers to differences in a particular observer's score on a patient that are not part of a systematic difference. To reduce these differences and to conduct more objective analyses, a lot of research has been conducted on digital image cytometry. This method mainly uses two-dimensional (2D) digital images to measure various characteristics of an object and the quantified characteristics can aid in the diagnosis and estimation of the prognosis of the cancer. However, these methods are not sufficient to quantify 3D structures. First, it is difficult to confirm the precise shape of a cell. For example, cells and cell nuclei are not perfectly spherical, and consequently, their shape differs noticeably depending on the cutting angle and the thickness of the sample. And the practical measurement is tedious, fatiguing, and time-consuming. To improve reproducibility, we need a new method, based on 3D image analysis. The 3D-based approaches have potential advantages over 2D-based approaches since the underlying tissue is 3D, thus making improved reproducibility and objectivity possible. From a hardware perspective, we can resolve the problems with 2D methods using a confocal microscope and image analysis techniques [7, 8], which can obtain successive 2D slices without physical sectioning. The image analysis techniques can be applied to volumetric data that has been reconstructed from the image slices obtained from the confocal microscope. From a methodological perspective, the measurement elements of the computer-based digital image analysis system are broadly divided into morphologic features and texture features [9, 10]. Morphologic analysis is conducted on the external aspects of the object, such as size, surface changes, length, and the ratio of long and short axes. Texture analysis quantifies 3D structures through a numerical analysis of changes in patterns, intensities, and other features in the image area.

Texture analysis has a long history, and a wide variety of methods have been studied and proposed in the past [11–14]. The gray level cooccurrence matrix (GLCM) is recognized as the most representative algorithm in spatial texture-related research. In particular, there are many recent published studies on how 3D GLCM expands conventional 2D GLCM methods. Kovalev et al. presented 2 models to characterize texture anisotropy in 3D MRI images [15]. One of the models is the intensity variation measure approach, which calculates a 3D GLCM and extracts a set of features from the histogram to describe the texture properties. Kurani et al. applied a 3D GLCM to organs of the human body in computed tomography (CT) images [16]. After extracting 10 texture features, they investigated the distribution characteristics of volumetric data for each organ.

Wavelets have also been researched for many years and are used in a wide variety of applications, including image compression and preprocessing. In addition, many studies have extracted texture values from images using wavelets and used them for classification [17–20].

As with the GLCM, recent studies on wavelet textures have also expanded from 2D to 3D wavelets. Yoo et al. extracted texture feature values from spaceborne images, for topographical sorting, by applying 6 3D wavelet basis functions, and reported a classification accuracy of 89% or higher [21]. However, they used wavelet coefficients for classification directly, without any definition and extraction of specific texture parameters. And their method has a low degree of stability because the classification accuracy dropped by between 20% and 40% on 2 of 5 segmented regions. Gao et al. evaluated the performance of 3D texture features for 3D MR brain image retrieval based on various approaches [22]. In that study, they used 4 types of methods: 3D local binary pattern (LBP), 3D GLCMs, 3D wavelets, and 3D Gabor textures. They compared the data retrieval performance on 100 volumetric data sets, with both normal and lesion brains, after measuring the similarity of the texture of the input data and data stored in a database. Although they used various types of 3D texture extraction methods, including 3D wavelet textures, the best results they achieved were 65%. Several previous studies used 3D wavelet transforms in image classification but most studies applied them to high-resolution images in which the characteristics of the objects are clearly distinguished. Our research materials with images of renal carcinoma have been subjectively graded by 2 pathologists. The main purpose of this study is to evaluate the validity of three-dimensional texture methods, which will be used to develop a computer-based image analysis system capable of automatically grading renal cell carcinoma tissue images.

## 2. Materials and Methods

### 2.1. Clinical Specimens and Image Acquisition

We obtained RCC tissue from 8 cases from the Department of Pathology, Yonsei University, Korea. They had been fixed in 10% neutral-buffered formalin and embedded in paraffin before receipt. The tissues were cut into 20-*μ*m sections, stained with propidium iodide (PI) containing RNase A at a final concentration of 0.5 mg/mL, and mounted in a fluorescent mounting medium (DAKO, Carpinteria, CA, USA). PI is a fluorescent dye that is often used to inspect cell nuclei and measure apoptosis. PI enables one to determine the content or state of the DNA of measured cells because it combines with DNA.

The RCC tissues were imaged under a TCS SP2 AOBS confocal laser scanning microscope (Leica Microsystems, Mannheim, Germany), with 630x zoom, a 1.4 NA HEX PL-Apochromat objective lens, and a HeNe laser. The fluorescently labeled antibody and PI were imaged simultaneously using the 468 and 568 nm laser lines. The light emitted between 596 and 685 nm fluorescein signal was collected with one photo multiplier tube (PMT), and the light of wavelengths 596~685 nm (PI signal) was collected with another PMT. The image regions of processing were defined by 2 pathologists. We obtained 30–120 slices for each volumetric data set; each slice was a 512 × 512 image with a resolution of RGB 24 bits/pixel. The voxel size of the data was 0.34 *μ*m in the *x* and *y* directions and 0.5 *μ*m in the *z* direction. However, appropriately downsampled images (256 × 256, RGB 24 bits/pixel) were used for final feature extraction and analysis with our developed software.

### 2.2. Preprocessing

**Figure 1:**
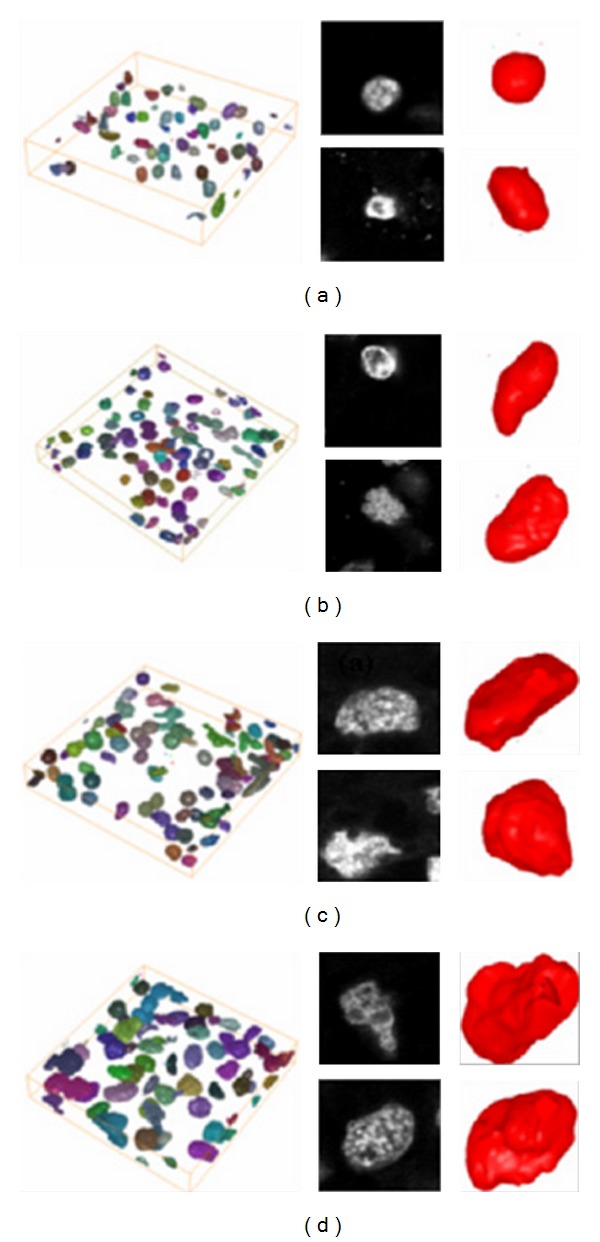
The representative 3D renal cell carcinoma (RCC) of confocal microscopic images (a) grade 1, (b) grade 2, (c) and (d) grade 3 and grade 4, respectively.

Noise in tissue images is generally caused by differences in the degree of dyeing, depending on the tissue thickness, and other external factors. When using a filtering method for medical image data, image degradation caused by blurring or artifacts resulting from a filtering scheme is not acceptable. To minimize these effects from the images, we applied bilateral filtering in 2 dimensions [23, 24]. It combines gray levels or colors based on both their geometric closeness and photometric similarity and prefers near values to distant values in both domain and range. It involves a weighted convolution in which the weight for each pixel depends not only on its distance from the center pixel but also on its relative intensity. In Figure 1, our developed software tool shows the representative 3D RCC visualization of the confocal microscopic images.

### 2.3. 3D GLCM and 3D Wavelet

Two-dimensional texture features were computed using pixels from each slice. However, if we were to process 3D volume data as individual 2D slices, then some interslice information would be ignored, increasing the possibility of data loss. To resolve this problem, we applied the concept of 3D texture features. Despite the simplicity of extending conventional matrix-based algorithms to 3 dimensions, this approach yielded significantly improved results.

**Figure 2:**
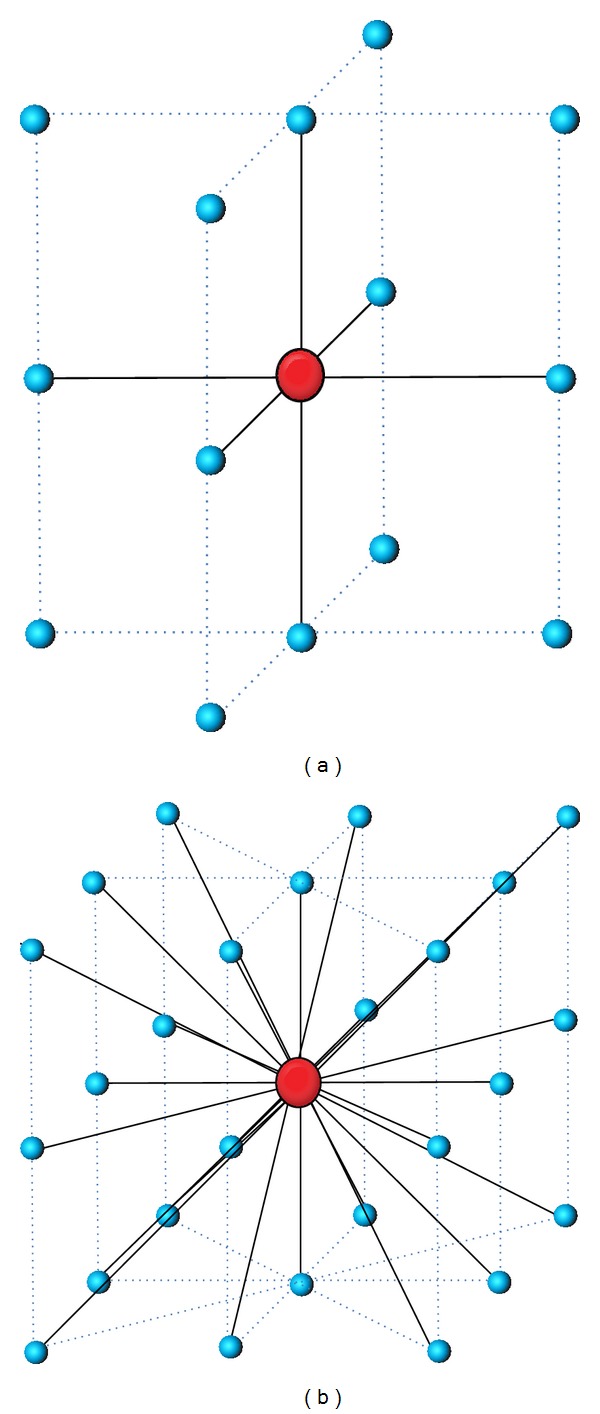
The spatial relationship of neighboring voxels in 3D. (a) 90° and (b) 45°.

The 2D GLCM considers the spatial dependency of pixels on one slice, while 3D GLCM quantifies the 3D dependency of voxel data on the object volume, which exists across several slices. Similar to the case of 2D, cooccurrence matrices for volume data also represent an *n* × *n* matrix in which *n* is the gray level. These matrices are defined using the specific displacement vector *d* = (*dx*, *dy*, *dz*) for each direction, where *dx*, *dy*, and *dz* are the number of voxels to move along the *x*-, *y*-, and *z*-axes, respectively, between samples. Pixels in 26 directions can be examined, but to avoid redundancy, only 13 directions were considered. We calculated 13 matrices for each data set, and then calculated a new matrix by averaging the 13 matrices. From this new matrix, we extracted 3D texture features.  Figure 2 shows the relationships among neighboring voxels for a central voxel with 6 neighbors when rotating 90° and 26 neighbors when rotating 45° between adjacency directions from the volume data.

Wavelet transform provides a spatial/frequency representation of a signal. Wavelet coefficients of a signal are the projections of the signal onto the multiresolution subspaces where the basis functions are constructed by dyadic dilations and translations of the scaling and wavelet functions. Having obtained the coefficients at a specific level *j*, we can calculate the coefficients at level *j* − 1 using a filter bank. Wavelet decomposition of a 2D signal can be achieved by applying 1D wavelet decomposition along the rows and columns of the image separately [25]. Similarly, wavelet transform of a 3D signal can be achieved by applying the 1D wavelet transform along all three directions.

**Figure 3:**
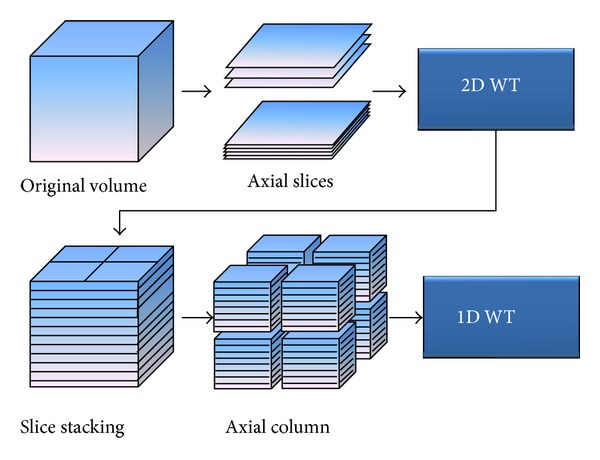
2D + 1D scheme for 3D wavelet transform.

The structural aspects of 3D wavelet transform have been defined and introduced in various ways. In this paper, we performed level 1 subband partitioning and generated 8 octant subbands using the “2D + 1D scheme” proposed by Chen and Ning [26] (see Figure 3). In this scheme, the 2D wavelet transform is first applied to each slice of a given data volume, and slice stacking is performed.

Then, the 1D wavelet transform is performed along the *z*-axis (axial). Originally, this scheme was proposed to extract a signal from a mixture of signal and noise (denoising) on breast cone-beam computed tomography (CBCT) volumetric data, and it included 2 filtering processes by coefficient modification as intermediate phases. However, in this research, we used a modified scheme for 3D texture extraction by skipping these processes.

**Figure 4:**
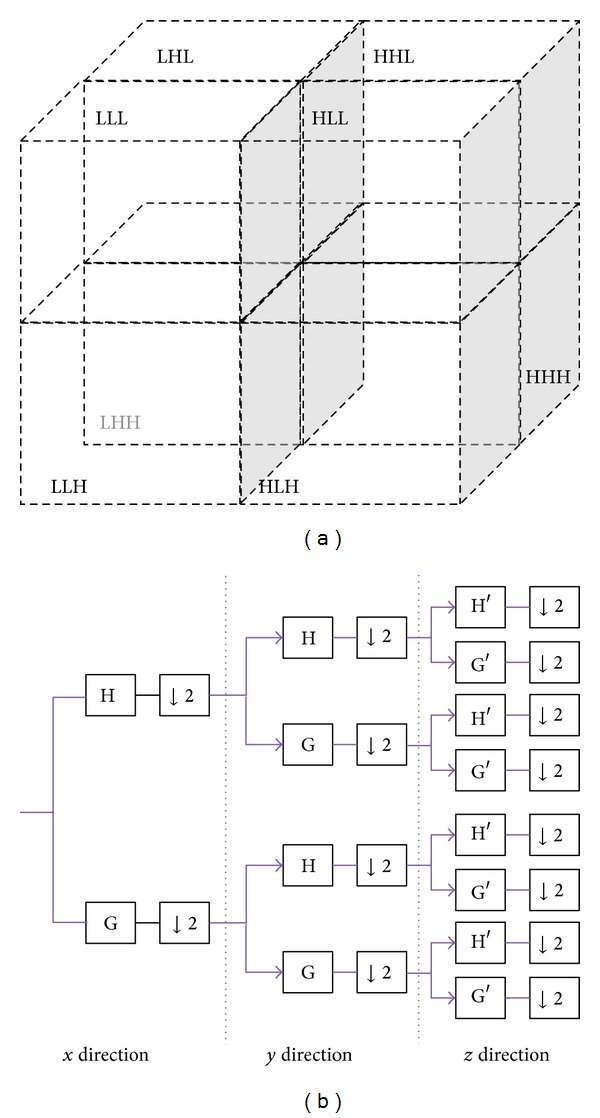
The concept of the wavelet decomposition in 3D and the structure of the filter bank corresponding to the 3D wavelet transform. (a) 3D wavelet octband decomposition (level 1) (b) filter bank.

Figure 4 shows a conceptual diagram of 3D wavelet decomposition and a filter bank. H and G and H′ and G′ are 2 different sets of filter banks. To determine the filter coefficient, this study used 2 wavelet basis functions: DB2 (Daubechies wavelet 2) and Haar (Haar wavlet).

The following are the reasons we have selected the two wavelets [27]. The Haar wavelet is the simplest and the shortest type of orthonormal wavelet. It is advantageous when pixels change dramatically and the multispectral image does not have many bands. The disadvantage of the Haar wavelet is that it is not continuous and therefore not differentiable. DB2 is a type of Daubechies wavelet, the most popular type of wavelet, which represents the foundation of wavelet signal processing and is used in numerous applications. Unlike other wavelets, most Daubechies wavelets are not symmetrical. The asymmetry of some Daubechies wavelets is very pronounced. Regularity increases with order, and the analysis is orthogonal.

### 2.4. Feature Extraction

To analyze tissue texture feature values and to facilitate grade classification, this study defined and extracted 12 feature values from the 3D GLCM and 16 feature values from the 3D wavelets, using 2 from each of the 8 subbands. The Appendix lists all of the feature values and formulas. The 12 feature values of the 3D GLCM are well known and can be calculated with the basic GLCM proposed by Haralick [28]. These 12 feature values were extracted by calculating independent matrices for 13 directions and then creating a new matrix with the averages of each matrix value.

For the 3D wavelets, 2 feature values, energy and entropy, were calculated for each band, resulting in 16 feature values for one data set. The wavelet energy features reflect the distribution of energy along the frequency axis over scale and orientation and have proven to be very effective for texture characterization. Because most relevant texture information is removed by iterative low-pass filtering, the energy of the low-resolution image is generally not considered a texture feature. Entropy can be interpreted as a measure of uncertainty, variability, and complexity. Entropy reaches its maximum in a completely random state and its minimum in a state of certainty. As a result, a homogeneous region produces the maximum entropy.

### 2.5. Statistical Analysis

After we extracted the feature values, we performed the statistical analysis [29–32]. We used 6 classifiers, defined according to their selection method, for the feature values and the type of the feature values.  Table 1 describes the characteristics of each classifier for tissue level analysis in detail.

For classifiers A, C, and E, a sequential stepwise method was used to select feature values. Stepwise selection combines forward and backward selection, repeating the addition and the removal of a feature at each step. This method can overcome the nesting problem (once a feature is added or removed, the decision cannot be changed). To evaluate the significance of a set of features, we needed some selection criteria. We used Wilk's lambda (*λ*) and the misclassification rate. Wilk's lambda is the ratio of the determinants of the within-class to the total covariance matrices and can be expressed as

(1) $$\begin{array}{c} \lambda =\frac{\left|W\right|}{\left|B+W\right|},\\ W=\underset{i}{\sum }\underset{j}{\sum }(x_{ij}-{\overset{-}{x}}_{i}){\left(x_{ij}-{\overset{-}{x}}_{i}\right)}^{\prime },\\ B+W=\underset{i}{\sum }\underset{j}{\sum }(x_{ij}-\overset{-}{x}){\left(x_{ij}-\overset{-}{x}\right)}^{\prime }, \end{array}$$

where *B* and *W* represent the between-class covariance matrix and the within-class covariance matrix, respectively. If the ratio of generalized variance is too small, the hypothesis that populations are identical is rejected, so a good set of features has a low Wilk's lambda value. From this process, we obtained 3 to 8 candidate features as the best candidates for grading.

Another selection approach is the principal component analysis (PCA), which was applied to classifiers B, D, and F [29, 30]. PCA is a method of reducing the dimensionality of a data set by finding a projection of the original set of vectors onto a lower-dimensional space, optimal in a mean-square sense. Based on the result of this analysis, we selected 8 principal components from the 3D wavelets and 5 components from the 3D GLCM by means of PCA and a correlation matrix. New feature values were defined and calculated from a linear combination of the originally extracted feature values and the unique values of each principal component.

After the candidate feature values were selected, we conducted a linear discriminant analysis (LDA) to generate a classifier. Discriminant analysis finds independent variables that can explain the difference between classes and generate a discriminant by using a combination of the variables.

The classification steps we followed using discriminant analysis are as follows: first, we organized a training data set and then performed a training process to improve classification accuracy. Then, we classified the rest of the data, except for training data, using each classifier. The amount of training data, the number of test data sets, and the number of slices per set used in this study are summarized in Table 2.

## 3. Results and Discussion

### 3.1. S/W Development and Test Environment

A computer system with an Intel Pentium 4 3.0 GHz processor and an NVIDIA GeForce 6800XT graphics card was used for software implementation and the performance test. All of the 3D feature values were automatically extracted using MATLAB version 6.5 release 13, with SP1 (Mathworks Inc., Novi, MI, USA), in accordance with the predefined format. Furthermore, all statistical analyses were conducted using SAS version 9.1.3 (SAS Institute Inc. Cary, NC, USA) at a significance level of 0.01 (99%).

**Figure 5:**
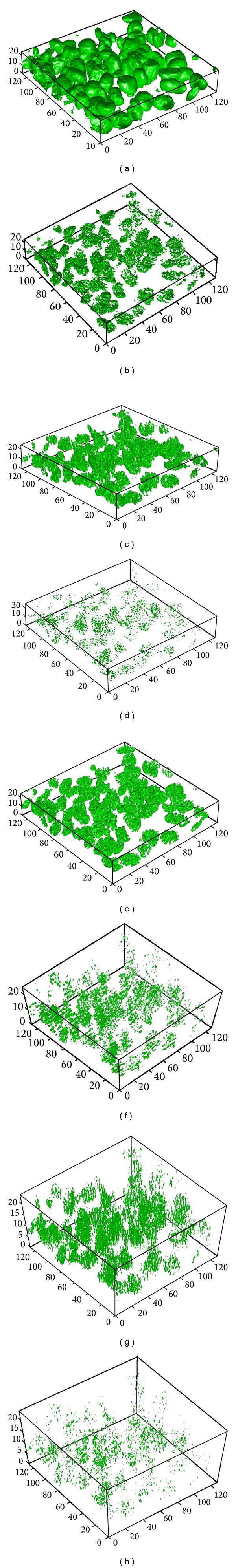
Visualization of the 8 octbands using iso-surface rendering (iso-value = 10). (a) LLL, (b) LLH, (c) LHL, (d) LHH, (e) HLL, (f) HLH, (g) HHL, and (h) HHH.

Figure 5 shows the grade 4 data used in this study, which were broken up into 8 octbands through the 3D wavelets and visualized by iso-surface rendering using MATLAB. To clearly visualize the differences between the bands, the iso-value was set to 10 for all bands. The differences between bands are clearly visible, as shown in this example, in which some bands include more texture characteristics than others. In other words, low-pass subbands usually contain most of the important information on the object, whereas noise and irregular characteristics are spread across most of the high-pass bands. Therefore, these characteristics can be quantified with more objective data through texture analysis.

### 3.2. Candidate Feature Selection

PCA and stepwise feature selection were carried out separately for each classifier before actual classification to find the feature values and use them for classification. The results of stepwise selection for classifiers A, C, and E are shown below. The *F*-value is the ratio of the mean square due to regression to the mean square due to error and indicates the influence (significance) of each controlled factor on the tested model. In the case of the Haar wavelet (A), we first selected 5 features, as shown in Table 3. Although Wilk's lambda values are low in most cases, 2 of 5 features are outside of the valid significance level of 0.01% (Pr > *F*). For this reason, only WEN_7 (HHL band), WEN_2 (LLH band), and WET_4 (LHH band) are used as independent variables for discriminant analysis. Similarly, in the case of the DB2 wavelet, which we describe in Table 4, we only used one of the eight selected features. In the case of the 3D GLCM (E) in Table 5, 3 features are selected as independent variables for discriminant analysis (variance (VAR), second-order diagonal moment (SDM), and second-order inverse difference moment (SIDM)).

We also applied PCA to the other three classifiers, B, D, and F. Initially, the number of principal components was set to be identical to the number of original feature values, and the number of principal components was determined on the basis of classifier explainability for both 3D wavelet and 3D GLCM. For classifiers B and D, we selected 8 of the 16 principal components, and for classifier F, we selected 5 of the 12 principal components.

Tables 6, 7, and 8 show the results of the principal components analysis for each classifier. In each table, the eigenvalue is an index of the degree to which each factor explained the original variables, and the sum of eigenvalues is equal to the number of variables. “Proportion” means a total variance, which is explained by a factor, and “cumulative” refers to accumulated values of proportion. In this research, we selected the number of principal components by inspecting these cumulative values.

In the case of classifiers B and D, we selected 8 principal components, which have a 95.59% and 95.41% degree of explanation, respectively. In the case of classifier F, we selected 5 principal components, which have a 99.46% degree of explanation.

### 3.3. Classification

The feature values selected through the above process were used for classification. First, the data were divided into training data and test data for classifier training. We used a preperformed training process to improve the correctness of the classification by using training data sets. For the training data, we randomly selected 4, 12, 7, and 11 data sets for each of the grades from 1 to 4, respectively. The average classification accuracy of the training data was 100% (A), 84.75% (B), 95.50% (C), 99.00% (D), 99.50% (E), and 91.25% (F). All other data sets, except for the training data, were used as test data. The accuracy of classification represents for each classifier using the training data sets.

After the training process for each classifier, we performed actual classifications by using the test data sets. Classifiers A and B used Haar wavelet basis functions. For classifier A, 3 candidate feature values were used for classification using stepwise feature selection. The results of classifier A were 75.00% for grade 1, 100.00% for grade 2, 66.66% for grade 3, and 72.73% for grade 4. The overall classification accuracy was approximately 81.25%. However, even though the candidate feature values were used in both cases, their discrimination between grades 2 and 3, which is known to be clinically difficult, was somewhat worse than expected.

Classifier B performed a classification using 8 principal components as candidate features. Of the six classifiers used in this experiment, classifier B showed the best discrimination results. It showed 100.00% for grade 1, 81.82% for grade 2, 100.00% for grade 3, and 90.91% for grade 4. The overall classification accuracy was 90.63%. To summarize, the classification accuracy of stepwise selection was high, and PCA was the most accurate with Haar wavelet basis functions.

Classifiers C and D used DB2 wavelet basis functions. In the case of classifier C, we selected one feature, which we used for classification. The test results for classifier C were 100.00% for grade 1, 45.44% for grade 2, 66.67% for grade 3, and 72.73% for grade 4. The overall classification accuracy was 65.63%. Classifier D used 8 principal components as candidate features, as was the case for classifier B. The test results of classifier D, which used DB2 as the basis function, were 75.00% for grade 1, 100.00% for grade 2, 66.67% for grade 3, and 45.45% for grade 4. The overall classification accuracy was 71.88%. These results were lower than those of classifier C, which used the same basis function.

The usefulness of 3D GLCM was evaluated using the same two approaches. It was expected that the validity of 3D GLCM would be high because the accuracy of grade classification at the nucleus level was high. However, both cases showed very low classification accuracy. Classifier E used 3 selected features for classification. Its classification accuracy was 25.00% for grade 1, 27.27% for grade 2, 50.00% for grade 3, and 90.91% for grade 4, and the overall classification accuracy was 53.13%. In this case, all grade 1 data were misclassified as grade 3 or 4, and variation among grades was the highest.

**Figure 6:**
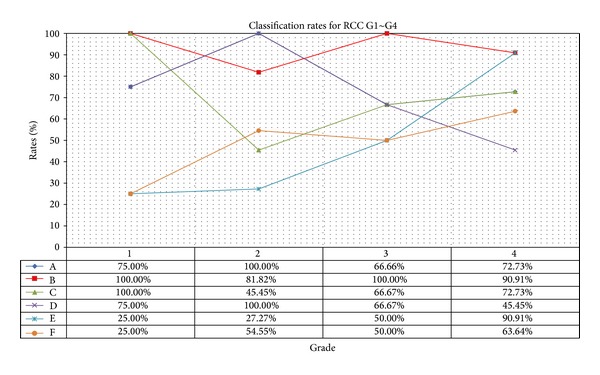
Comparison of classification performance using 6 different models.

Classifier F, which applied PCA, showed similar results. Its classification accuracy was 25.00% for grade 1, 54.55% for grade 2, 50.00% for grade 3, and 63.64% for grade 4. The overall classification accuracy was 53.13%. Again, the classification of grade 1 data was generally incorrect. Both classifiers showed high classification accuracy for the training data, but the results were lower than 50% for the test data. This may be caused by the fact that the data extraction allows many degrees of freedom, which results in overtraining on the training data because of the low number of data sets we have available.  Figure 6 compares the classification results for the six models using the test data. The statistical analysis results indicated that a considerable amount of data was misclassified into grade 2. However, classifier B solved a considerable portion of this problem and showed the most stable accuracy when compared to the other five classifiers. The results were confirmed by 2 pathologists and the correlation study between the subjective and computerized grading.

By using 3D texture features, our scheme classifies the renal cell carcinoma tissue images with reasonable accuracy into 4 grades. A total of 28 features were carefully chosen from 2 categories of texture features. In order to confirm the validity of 3D texture features, we tested the ability of each to discriminate between the four grades based on statistical analysis in diverse forms. One of the biggest problems in previous studies has been that the extraction of individual objects for nucleus unit processing takes a considerable amount of time [9]. In this respect, this study focused on the extraction and classification of texture feature values and processed data at the tissue level. Generally, in image classification, it is clear that there is no best texture set. Instead, the best results come from a combination of several texture parameters, which complement one another. However, it is impractical to investigate all possible combinations of all possible subsets in order to find this best combination.

## 4. Conclusions

Texture analysis in pathological images is a complex task requiring appropriate textural descriptors that reflect the biological properties of the pathological materials. The aim of this study was to develop a new software approach to analyze 3D texture features in order to detect subtle changes in thick cancer tissue section images by confocal laser scanning microscopy. This study is differentiated from previous works in pathological image grading in that we performed actual classifications by using 3D texture features and investigated the validity of these classifications. Therefore, we used stepwise feature selection and PCA techniques to find the best candidate features. We expected both of the two types to perform well because we had already confirmed the validity through our preliminary study [33]. However, the discriminant power was less than expected. In particular, the results of 3D GLCM did not meet our expectations. We found that it is difficult to apply because it showed very low accuracy in the tissue level tests. Unlike the preliminary study, which extracted feature values by considering individual objects (i.e., cell nuclei), tissue unit images require more precise feature values because various subcellular objects coexist in one image. In our tests, classification using 3D wavelet texture features was better than that of 3D GLCM. In particular, the classification accuracy of Haar wavelets and PCA was better than 90%, and these two showed an even distribution among the different grades. Another interesting finding is that Haar wavelets showed higher accuracy than DB2, contrary to our expectations. It is generally known that longer wavelets are more accurate than Haar wavelets for the classification of high-resolution images. However, it had exactly the opposite effect in our case. The results of this study point in several promising directions for future research and could be a valuable tool for further developments in the field of tissue analysis.

Although the results need to be verified in larger studies, this type of quantification using 3D texture feature values has a potential for the development of computer-based image grading systems for more accurate diagnosis and prognosis. Furthermore, we believe that 3D textures can be utilized in various areas as a useful tool for the processing and analysis of medical imaging data, as well as the extraction of feature values for classification.

## Figures and Tables

**Table 1:** Description of the six classifiers.

Classifier	Texture	Feature selection method	Number of features
A	3D Wavelet (Haar)	Stepwise selection	16
B	3D Wavelet (Haar)	PCA	16
sC	3D Wavelet (DB2)	Stepwise selection	16
D	3D Wavelet (DB2)	PCA	16
E	3D GLCM	Stepwise selection	12
F	3D GLCM	PCA	12

**Table 2:** A summary of the data set for each grade.

Grade	Number of train data sets	Number of test data sets	Minimum number of slices	Maximum number of slices
I	4	4	47	99
II	12	11	37	98
III	7	6	40	78
IV	11	11	37	126

**Table 3:** Stepwise selection result for classifier A (Haar).

Step	Entered	*F*-Val.	*Pr*⁡>*F*	Wilks' Lambda	Pr < Wilks' Lambda
***1***	***WEN_7***	***22.14***	***<0.0001***	***0.29658280***	***<0.0001***
***2***	***WEN_2***	***18.11***	***<0.0001***	***0.22915727***	***<0.0001***
***3***	***WET_4***	***14.59***	***<0.0001***	***0.16365931***	***<0.0001***
4	WET_2	8.18	0.0158	0.11035852	<0.0001
5	WEN_3	2.02	0.1382	0.03576547	<0.0001

**Table 4:** Stepwise selection result for classifier C (DB2).

Step	Entered	*F*-val.	*Pr*⁡>*F*	Wilks' lambda	Pr < Wilks' lambda
***1***	***WEN_2***	***35.26***	***<0.0001***	***0.20929053***	***<0.0001***
2	WET_7	8.92	0.0003	0.10511490	<0.0001
3	WET_6	3.83	0.0214	0.07289270	<0.0001
4	WEN_6	3.67	0.0256	0.05060370	<0.0001
5	WET_1	2.90	0.0555	0.03712685	<0.0001
6	WEN_1	2.92	0.0559	0.02689915	<0.0001
7	WEN_4	2.60	0.0779	0.01986165	<0.0001
8	WEN_8	2.05	0.1375	0.01536082	<0.0001

**Table 5:** Stepwise selection result for classifier E (GLCM).

Step	Entered	*F*-Val.	*Pr*⁡>*F*	Wilks' lambda	Pr < Wilks' lambda
***1***	***VAR***	***47.38***	***<0.0001***	***0.40614582***	***<0.0001***
***2***	***SDM***	***21.37***	***<0.0001***	***0.35268221***	***<0.0001***
***3***	***SIDM***	***4.19***	***<0.0001***	***0.21780024***	***<0.0001***

**Table 6:** The explanatory adequacy of principal components for classifier B (Haar).

Eigenvalues of correlation matrix				
	Eigenvalue	Difference	Proportion	Cumulative
*1 *	*11.2629567 *	*10.0532493 *	*0.7039 *	*0.7039 *
*2 *	*1.2097075 *	*0.4309052 *	*0.0756 *	*0.7795 *
*3 *	*0.7788023 *	*0.1339744 *	*0.0487 *	*0.8282 *
*4 *	*0.6448297 *	*0.1606645 *	*0.0403 *	*0.8685 *
*5 *	*0.4841633 *	*0.0898111 *	*0.0303 *	*0.8988 *
*6 *	*0.3943523 *	*0.0843736 *	*0.0246 *	*0.9234 *
*7 *	*0.3099786 *	*0.1003198 *	*0.0194 *	*0.9428 *
***8***	***0.2096588***	***0.0385356***	***0.0131***	***0.9559***
9	0.1711232	0.0161924	0.0107	0.9666
10	0.1549309	0.0380616	0.0097	0.9763
11	0.1168693	0.0266911	0.0073	0.9836
12	0.0901782	0.0154420	0.0056	0.9892
13	0.0747362	0.0120109	0.0047	0.9939
14	0.0627253	0.0373784	0.0039	0.9978
15	0.0253469	0.0157044	0.0016	0.9994
16	0.0096425		0.0006	1.0000

**Table 7:** The explanatory adequacy of principal components for classifier D (DB2).

Eigenvalues of correlation matrix				
	Eigenvalue	Difference	Proportion	Cumulative
*1 *	*11.4562631 *	*10.4538753 *	*0.7160 *	*0.7160 *
*2 *	*1.0023878 *	*0.2936114 *	*0.0626 *	*0.7787 *
*3 *	*0.7087764 *	*0.0289652 *	*0.0443 *	*0.8230 *
*4 *	*0.6798112 *	*0.1316535 *	*0.0425 *	*0.8655 *
*5 *	*0.5481576 *	*0.2369534 *	*0.0343 *	*0.8997 *
*6 *	*0.3112042 *	*0.0156132 *	*0.0195 *	*0.9192 *
*7 *	*0.2955911 *	*0.0317010 *	*0.0185 *	*0.9376 *
***8***	***0.2638901***	***0.0836416***	***0.0165***	***0.9541***
9	0.1802484	0.0120338	0.0113	0.9654
10	0.1682147	0.0587713	0.0105	0.9759
11	0.1094434	0.0260516	0.0068	0.9827
12	0.0833918	0.0045418	0.0052	0.9880
13	0.0788500	0.0265018	0.0049	0.9929
14	0.0523481	0.0134512	0.0033	0.9962
15	0.0388969	0.0163717	0.0024	0.9986
16	0.0225252		0.0014	1.0000

**Table 8:** The explanatory adequacy of principal components for classifier F (GLCM).

Eigenvalues of correlation matrix				
	Eigenvalue	Difference	Proportion	Cumulative
*1 *	*9.20978568 *	*7.47985153 *	*0.7675 *	*0.7675 *
*2 *	*1.72993416 *	*1.22514588 *	*0.1442 *	*0.9116 *
*3 *	*0.50478828 *	*0.20578467 *	*0.0421 *	*0.9537 *
*4 *	*0.29900361 *	*0.10748828 *	*0.0249 *	*0.9786 *
***5***	***0.19151532***	***0.14235080***	***0.0160***	***0.9946***
6	0.04916452	0.03985567	0.0041	0.9987
7	0.00930885	0.00518274	0.0008	0.9995
8	0.00412611	0.00277631	0.0003	0.9998
9	0.00134980	0.00049960	0.0001	0.9999
10	0.00085020	0.00067674	0.0001	1.0000
11	0.00017346	0.00017346	0.0000	1.0000
12	0.00000000		0.0000	1.0000

**Table 9:** The list of the 3D texture features extracted.

Features	Equations
3D GLCM^1^	
Angular second moment (also called energy)	$\text{ASM}=\underset{i}{\sum }\underset{j}{\sum }{c(i,j)}^{2}$
Entropy	$\text{ENTR}=\;-\underset{i}{\sum }\underset{j}{\sum }c\left(i,j\right)*\log\left(c\left(i,j\right)\right)$
Correlation	$\text{CORR}=\underset{i}{\sum }\underset{j}{\sum }\frac{\left(i*j\right)*c\left(i,j\right)\;-\left(\mu_{x}*\mu_{y}\right)}{\sigma_{x}*\sigma_{y}}$
Contrast	$\text{CONT}=\;\overset{G-1}{\underset{i-j=0}{\sum }}{\left(i-j\right)}^{2}\overset{G}{\underset{i=1}{\sum }}\overset{G}{\underset{j=1}{\sum }}c(i,j)$
Variance	$\text{VAR}=\overset{2N}{\underset{i,j}{\sum }}{\left(i-\mu_{x}\right)}^{2}c(i,j)$
Sum mean	$\text{SM}=\overset{2N}{\underset{i,j=1}{\sum }}ic_{x+y}(i)$
Sum variance	$\text{SV}=\overset{2N}{\underset{i,j=1}{\sum }}{\left(i-\mu_{x+y}\right)}^{2}c_{x+y}(i)$
Cluster shade	$\text{CS}=\overset{N}{\underset{i,j=1}{\sum }}{\left(i-M_{x}+j-M_{y}\right)}^{3}c(i,j)$
Cluster tendency	$\text{CT}=\overset{N}{\underset{i,j=1}{\sum }}{\left(i-M_{x}+j-M_{y}\right)}^{4}c(i,j)$
Second-order inverse difference moment (also called homogeneity)	$\text{SIDM}=\underset{i}{\sum }\underset{j}{\sum }\frac{1}{1+{\left(i-j\right)}^{2}}c(i,j)$
Peak transition probability	$\text{PTP}=\underset{}{\max}(c\left(i,j\right))$
Second-order diagonal moment	$\text{SDM}=\sqrt{0.5*\left(j-i\right)c(i,j)}$
3D Wavelet^2^	
Energy for 8 subbands (WEN1~8)	$\text{WEN}=\overset{N}{\underset{i,j,k=1}{\sum }}{\left(W\left[i,j,k\right]\right)}^{2}$
Entropy for 8 subbands (WET1~8)	$\text{WET}=-\overset{N}{\underset{i,j,k=1}{\sum }}W^{2}\left[i,j,k\right]\log_{2}\left(W^{2}[i,j,k]\right)$

^1^c(i, j) represents a cooccurrence matrix. M_x  _ = ∑_i,j=1_^N^ic(i, j), M_y  _ = ∑_i,j=1_^N^jc(i, j).

^2^ *W*[*i*, *j*, *k*] represents a 3D matrix for a certain band.

## Appendix

See Table 9.
